# Case Report: Morphologic and Functional Characteristics of Intestinal Mucosa in a Child With Short Bowel Syndrome After Treatment With Teduglutide: Evidence in Favor of GLP-2 Analog Safety

**DOI:** 10.3389/fnut.2022.866048

**Published:** 2022-06-23

**Authors:** Enrico Costantino Falco, Antonella Lezo, Pierluigi Calvo, Caterina Rigazio, Anna Opramolla, Ludovica Verdun, Giovanna Cenacchi, Marianna Pellegrini, Marco Spada, Gabriella Canavese

**Affiliations:** ^1^Department of Pathology, AOU Città della Salute e della Scienza di Torino, Turin, Italy; ^2^Dietetics and Clinical Nutrition Unit, Children’s Hospital Regina Margherita, AOU Città della Salute e della Scienza di Torino, Turin, Italy; ^3^Department of Pediatric Gastroenterology, AOU Città della Salute e della Scienza di Torino, Turin, Italy; ^4^Department of Biomedical and Neuromotor Sciences, Alma Mater Studiorum Universitá di Bologna, Bologna, Italy

**Keywords:** teduglutide, short bowel syndrome, treatment, safety, proliferation

## Abstract

Teduglutide is a glucagon-like peptide-2 (GLP-2) analog employed in patients with short bowel syndrome (SBS) to reduce the need of parenteral nutrition in these patients, by virtue of its effects on enteric function. The experimental studies reported that the stimulating action of GLP-2 on epithelial turnover implies the potential development of dysplastic and neoplastic lesion. However, the clinical trials could not detect preneoplastic lesions on histologic material, and in a recent pilot study the occurrence of polyps was similar before and after treatment and included only low-grade dysplastic lesions. Another clue in GLP-2 function in stimulating mucosal restore is its enhancement through cooperation with epidermal growth factor (EGF). In this study, we analyzed gastroscopy and colonoscopy samplings from a child successfully weaned off parenteral nutrition with teduglutide. Villous and crypt structure was regular both in duodenal and in colonic samplings; in properly oriented villi, villus/crypt ratio was regular. The absorptive epithelium demonstrated a regular morphology. No atypia was detected in enterocytes, along epithelial structures. At the ultrastructural analysis, only a few enterocytes with vacuolized cytoplasm were observed. An S-phase marker Ki67 stained nuclei in the transitional amplifying zone, while nuclei stained by the cell cycle regulatory proteins p21 and p27 were placed in the differentiated epithelium of the duodenal villi and colonic crypts, as in the control cases. The counts of enterocytes immunostained with the same antisera, evaluated with image analysis software, were in the range of control cases. The ratio of the number of epidermal growth factor receptor (EGFR) signals/the number of centromere probe of chromosome 7 (CEP7) signals was less than 2. The findings available from this single patient are consistent with good preservation of functional capability of intestinal epithelium after treatment with GLP-2, given the histologic and ultrastructural features of enterocytes. In addition, the findings from cell cycle regulatory proteins immunolocalization and quantitative analysis show that cell renewal machinery in our case is comparable to control cases. The gene of the receptor EGFR is regularly expressed in enteric epithelium of our case. Morphologic and functional data from our patient improve evidence in favor of the safety of GLP-2 employ in SBS.

## Introduction

Short bowel syndrome (SBS) refers to a condition subsequent to the surgical resection of bowel defined as “the reduction of functional gut mass below the minimal amount necessary for digestion and absorption adequate to satisfy the nutrient and fluid requirements for maintenance in adults or growth in children” (1). The conditions most commonly leading to extensive small bowel resections in children are necrotizing enterocolitis (NEC), intestinal atresia, gastroschisis, and extensive aganglionosis in Hirschsprung disease. The clinical manifestations of SBS rely on the functional characteristics of the resected segment and on the ability of the residual tract to balance the loss of nutrient intake, more than on the length of the resected tract. Home parenteral nutrition (HPN) is the main treatment option for patients with SBS together with other measures aimed at promoting bowel adaptation and providing sufficient nutrition and growth. Survival of patients on HPN has improved dramatically during the last three decades, although management of treatment-related complications (catheter-related sepsis, liver disease, and loss of venous access) is still challenging (2).

A further step forward was taken in the nineties of the last century, when it was proved that glucagon-like peptide-2 (GLP-2), one of proglucagon-derived peptides (PGDPs), could prompt intestinal mucosa expansion through the increase of the proliferative activity of crypts, in conjunction with other effects as an increase in intestinal blood flow, intestinal barrier function, inflammatory insults resistance and fluid absorption (3). A subsequent study demonstrated that GLP-2 reduced diarrhea and improved intestinal wet weight absorption in patients with SBS (4). A GLP-2 analog, Teduglutide, was then developed to inhibit the proteolytic degradation of the native molecule, extending its biological half-life (5).

In a phase II trial, which was conducted on the patients with SBS treated with teduglutide, villus height and crypt depth of the patients (with SBS) with end jejunostomy were measured, using light microscopy on 10 well-oriented villi and crypts; furthermore, the number of mitotic figures per 100 crypt epithelial cells was calculated as follows: Teduglutide significantly increased villus height (*p* = 0.030), crypt depth (*p* = 0.010), and mitotic index (*p* = 0.010) (6). Another phase III trial (7) confirmed the results, showing that small bowel villus height and colonic crypt depth were significantly increased in patients treated with teduglutide compared with placebo. However, in a study on patients of the aforementioned trial that underwent biopsy, mucosal DNA concentrations (mg DNA/mg biopsy), an index of cellularity, was not increased in patients treated with teduglutide (8).

About the consequences of the stimulating action of GLP-2 on epithelial turnover, preliminary studies in rat models reported carcinogenetic effects (9–11). On the other side, the previously cited clinical trials could not detect dysplastic lesions on histologic material. In a recent pilot study, the occurrence of polyps before and after treatment with teduglutide was evaluated, which is discussed as follows: The colon polyps were reported at baseline in 12% of patients and post-exposure in 18% of patients, including only low-grade dysplastic lesions (12). To evaluate the safety of teduglutide in pediatric patients, the efficacy of teduglutide was tested in a neonatal piglet jejunostomy model and the study showed that immunohistochemical distribution of Ki67, was not affected by the treatment (13).

Given the lack of GLP-2 receptors in colonic enterocytes, the effects of the protein on enterocytes metabolism are driven by EGF/human epidermal growth factor receptor 2 (HER-2) receptors, though several evidence on the experimental models proved that the association of GLP-2 and EGF enhance the recovery of intestinal mucosa after parenteral nutrition (14–17).

In this study, we analyzed gastroscopy and colonoscopy samplings from a child treated with teduglutide, to evaluate the morphologic and ultrastructural features of the enteric mucosa. The integrity of the cell renewal machinery of the crypt was evaluated with immunohistochemistry as discussed in the following: the Ki67 was adopted as the most widely employed marker of all active phases of the cell cycle (G1, S, G2, and mitosis) (18–20). The p21 Kip-1 and p27 Waf-1 were chosen as inhibitors of proliferative pathways that allow differentiative processes in mature enterocytes (21, 22). The status of EGFR gene in intestinal enterocytes was defined with fluorescent in situ hybridization (FISH) technique. The goal of the investigation was the assessment of morphologic or functional anomalies referable to the increased risk of neoplasia in enteric mucosa of a patient treated with GLP-2.

## Materials and Methods

The patient was a 11-year-old girl with SBS after extensive bowel resection for congenital extensive intestinal atresia and a residual ultra-short bowel consisting of duodenum, 10 cm of terminal ileum, ileocecal valve, and whole colon. Thanks to the intestinal adaptation without HPN long-term complications; need for HPN progressively decreased. At 9 years of age, she was still on HPN; 4 infusions/week, 1,235 kcal/infusion, providing 35% of total energy need. Attempts to further reduce the parenteral nutrition (PN) failed since she developed recurrent metabolic acidosis and neuropsychiatric symptoms such as irritability and aggressiveness. The surgical options were not recommended as the intestinal magnetic resonance imaging (MRI) revealed no dilated segments. Teduglutide was started (0.05 mg/kg/die subcutaneously) in November 2018. At the follow-up visits, she showed decreased fecal output, increased diuresis as well as abundant oral intake, so PN was progressively reduced and stopped after 6 months of the therapy. After 2 PN-free years on teduglutide, no adverse events were observed, the patient gradually gained weight with a good tolerance to total oral feeding, normal laboratory tests and a better quality of life. Currently, the therapy with teduglutide is ongoing; the girl showed optimal compliance and growth rate with no need for PN support. Two attempts to temporarily suspend teduglutide failed as patient lost weight, increased fecal loses and newly developed acidosis, so after 2 weeks we have restored therapy.

No endoscopic samples of the pretreatment phase were available. The first gastroscopy and colonoscopy were performed in September 2020. Samplings from descending duodenum, duodenal bulb, stomach, residual ileum, ascending colon, transverse colon, sigmoid and rectum were taken, and two sampling from ileum and colon were fixed in glutaraldehyde for electron microscopy evaluation. Both duodenal and ileal sampling were oriented on acetate cellulose strips at the time of the biopsy.

Sections from small intestinal (ileum, bulb, and duodenum) and colonic samples (transverse colon) were employed for staining. Histochemical periodic acid schiff (PAS) stain was performed to highlight the distribution of glycogen, glycoproteins, and proteoglycans in cytoplasm and vacuoles of mucosal enterocytes. To define the cell renewal cycle of the intestinal mucosa, sections were immunostained with the following antibodies: KI67 clone Mib-1 Dako Agilent, dilution 1:50; p21 Waf-1, clone DCS-60.2; p27 Kip-1, clone SX53GB. The same immunostains were also performed on five duodenal biopsies (four pediatric patients and one adult) and four colonic samples from controls (two children and two adults), that demonstrated a regular morphology at histological examination. To quantify the number of immunoreactive enterocytes, stained nuclei were counted with an image analysis software an open-source Java image processing program (imagej.net) (23). The counts were performed on a 2-mm segment of duodenal and colonic mucosa with well-oriented crypts.

The sections from duodenal and colonic samples were employed for FISH hybridization. The case was tested with the probe mixtures set, that consists of two separate probes: EGFR (7p11) (Spectrum Orange)/CEP7 (7p11.1–7q11.1) (Spectrum Green) (Abbott Molecular, Roma, Italy). A 10 μl of the probe mixture was applied to one of the two slides and immediately covered with a coverslip and sealed with rubber cement. Slides were thoroughly co-denatured in the HYBrite System (Vysis) at 74°C for 5 min and overnight hybridized at 37°C. The slides were washed with 2 × SSC/0.3% NP40 washing solution at 73°C for 3 min, dehydrated, and counterstained with DAPI I (4,6-diamidine-2-phenylindol Vysis, Downers Grove, IL, United States).

Other samples from the small intestine and colon were fixed in 2.5% glutaraldehyde in cacodylate buffer, dehydrated in ethanol, and embedded in epoxy resin. Thin sections counterstained in uranyl acetate and lead citrate were studied under a Philips 410 transmission electron microscope.

## Results

**FIGURE 1:**
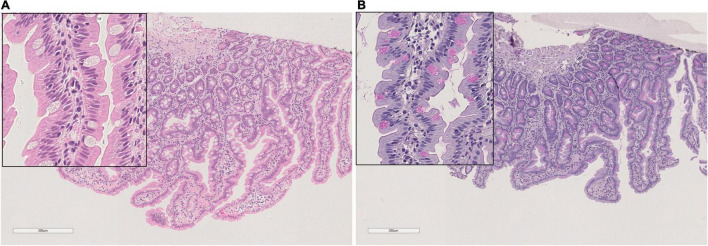
Duodenal mucosa: **(A)** Hem Eos, **(B)** PAS stain o.m. 40×.

**FIGURE 2:**
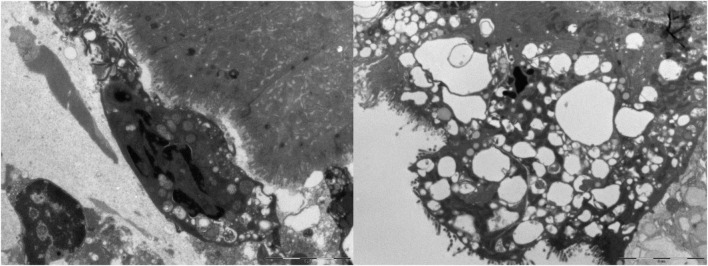
Ultrastructural image of ileal **(left)** and colonic **(right)** mucosa.

**FIGURE 3:**
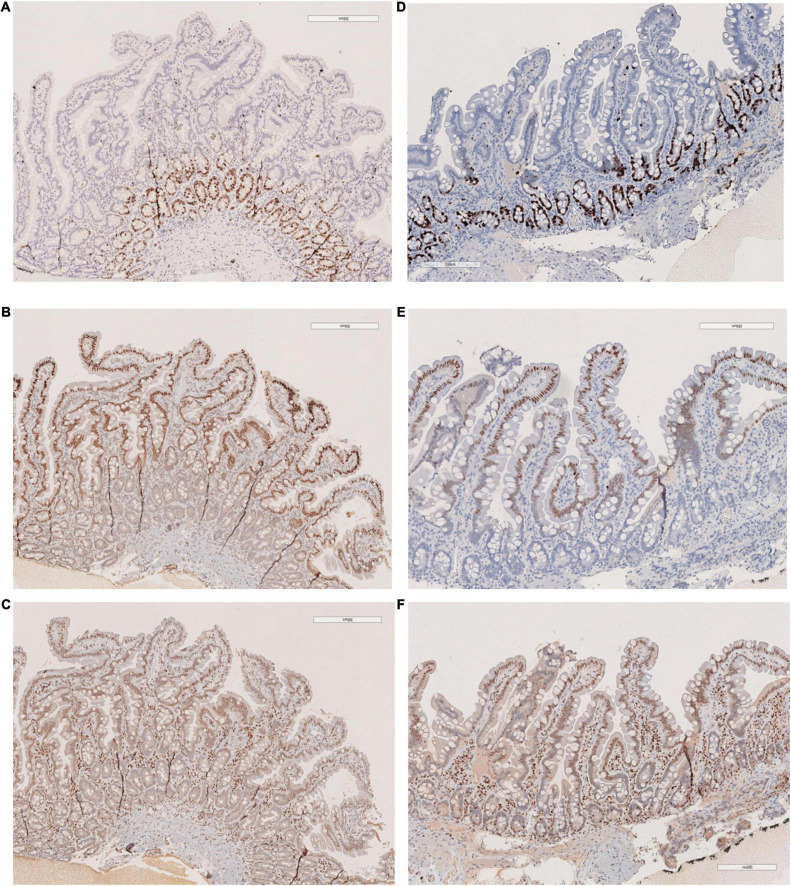
Duodenal mucosa: **(A)** KI67, **(B)** p21, **(C)** p27, Control case **(D)** KI67, **(E)** p21, **(F)** p27.

**FIGURE 4:**
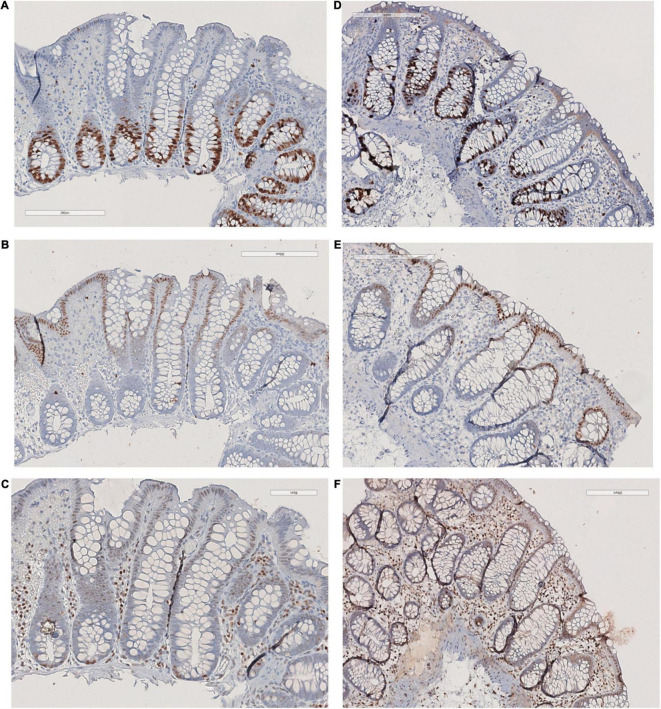
Colonic mucosa: **(A)** KI67, **(B)** p21, **(C)** p27; Control case: **(D)** KI67, **(E)** p21, **(F)** p27.

The villous structure was regular both in duodenal and ileal sampling, even if compression artifacts and inadequate orientation have restricted an accurate evaluation of villous length. However, in the properly oriented villi, villous/crypt ratio was within the normal range (between 1:3 and 1:4). Looking at the absorptive epithelium of small intestine, PAS staining demonstrated a correct morphology and distribution of goblet cells, and no irregular deposits of PAS positive mucins in enterocytes. The same staining proved the preservation of enterocyte brush border. Both in small intestinal and in large intestinal mucosa, no atypia was detected in enterocytes, along mucosal structures (Figure 1) and counts of crypt mitosis were not above 1 per crypt (0,75 mitotic figures/crypt in 20 well-oriented evaluated crypts), that is considered in the normal range when crypt hyperplasia is evaluated in the classification of celiac disease (21). White series elements in lamina propria consisted of mature lymphocytes and plasma cells, and their distribution and density were compatible with a normal resident infiltrate (Figure 1). The distribution of the immunostaining of the above-described regulators of cell renewal activity was evaluated. In all examined samples, Ki67 stained nuclei in S-phase were placed at the so-called transitional amplifying zone, while nuclei stained by the regulatory proteins p21 Kip-1 and p27 Waf-1 were placed in the differentiation district of the mucosal structures, that is the superficial epithelium of colonic mucosa and the villous epithelium of duodenal samplings. The localization of immunostained cells observed in the control cases was comparable to that described in our case. Figures 2, 3 show small and large intestinal samplings of our case, compared with a control case as an example. Data about counts of immunostained enterocytes, evaluated with image analysis software are resumed in Table 1. All counts regarding the reported case were in the range of the control cases, except for a slight exceed in p21 Kip-1 count in colon samples (50% of standard deviation). Counts of p27 Waf-1 on colonic samples were not performed for interference with stromal lymphocyte staining (Figure 4).

**TABLE 1:** Results of the counts on epithelial cells immunostained with Ki67 Mib-1, p21, and p27 with an image analysis software (see text) in small intestinal and colonic sampling, in our case and controls.

Small intestinal mucosa				

	Case	Controls (5 cases)		
		
	Count of stained nuclei	Median of stained nuclei	Range of stained nuclei	Standard deviation
Ki67	254	288	147–385	79
p21	1,017	1,083	702–1,230	222
p27	851	870	865–966	46

**Colonic mucosa**				

	**Case**	**Controls (4 cases)**		
		
	**Count of stained nuclei**	**Median of stained nuclei**	**Range of stained nuclei**	**Standard deviation**

Ki67	306	297	286–340	20
p21	364	286	247–348	35
p27	ND*	ND*		

*See text.

**FIGURE 5:**
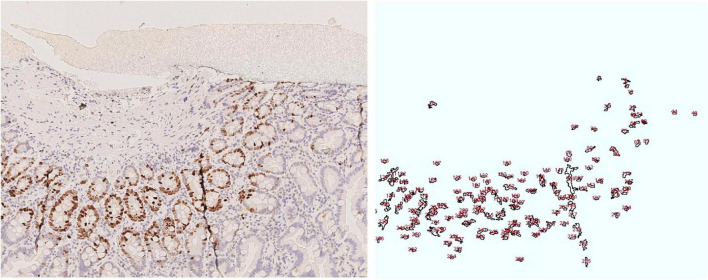
Duodenal mucosa, Ki 67 image and drawing of the image analysis. Cell nuclei were counted using the following parameters range as listed in the following: size (inches^2^) 20-infinity; circularity (0.10–1.00).

Ultrastructural analysis of small intestinal and colonic samples revealed a generally well-organized epithelial lining. Only a few enterocytes with vacuolized cytoplasm and degenerated epithelial cells shedding in the luminal space were observed. Lymphocytes, plasma cells, and eosinophil granulocytes were detected in the lamina propria (Figure 5).

**FIGURE 6:**
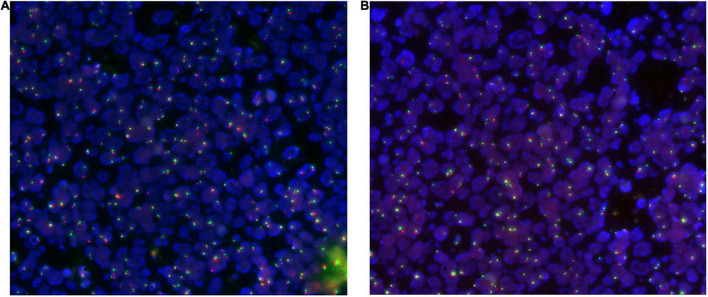
Duodenal **(A)** and colonic **(B)** tissue hybridized with the EGFR/CEP7 probe: nuclei are highlighted in blue, red signals instead highlight the EGFR gene (7p11) while green signals highlight the centromere of chromosome 7 (CEP7).

Both small intestinal and colonic tissue were hybridized with the EGFR/CEP7 probe. The EGFR gene was found, which was not amplified, since the ratio of the number of EGFR signals/the number of CEP7 signals was less than 2 (Figure 6).

## Discussion

Teduglutide is a GLP-2 analog employed in pediatric patients with SBS with the goal of reducing the need for parenteral nutrition in these patients, by virtue of its functional effects on enteric function as discussed in the following: Slowdown of gastric emptying, inhibition of gastric secretions, increase of intestinal blood flow, and proliferative activity. From the early studies, the trophic effect of GLP-2 on small and large intestinal mucosa was demonstrated in experimental models and clinical studies; an increase in small bowel villous height and intestinal crypt depth was documented in a series of clinical trials, together with an increase in mitotic activity. On the other side, the well-established stimulatory action of GLP-2 implies the potential risk of developing pre neoplastic and neoplastic lesions in enteric mucosa, and the results of the available studies on these topics are still debated, even if no increase in dysplastic or neoplastic lesions was described in clinical trials.

This study has the purpose of adding evidence on these topics, evaluating histopathological and immunophenotypic features of the enteric biopsies of a child with SBS successfully treated with teduglutide. Looking at the morphologic evidence, small and large intestinal samples showed villi and crypts with well-structured enterocytes, crypts with normal mitotic activity and regular villus/crypt ratio in the small intestinal mucosa after 22 months of teduglutide treatment. The ultrastructural profile of duodenal and colonic mucosa detected negligible alterations, with an uncertain correlation with SBS damage. Therefore, despite the absence of a pretreatment mucosal sampling, these finding, along with the considerable clinical improvement, are consistent with a complete restore of mucosal atrophy due to parenteral nutrition after teduglutide treatment. On the other hand, this study could not confirm the existing data about villous and crypt lengthening in patients treated with GLP-2 (6–8). In the routine samples, mechanical artifacts and specimen orientation make a precise measuring of these structures difficult, even if oriented on cellulose acetate strips, as demonstrated by studies on duodenal biopsies in patients with coeliac disease, that recommend quantitative morphometric analysis for a reliable evaluation of villous length on routine biopsies (24–27).

In the same biopsies, we evaluated the immunophenotypic expression of S-phase marker (KI67 Mib-1) and proliferative inhibitors (p21 Kip-1 and p27 Waf-1) in our patient and in a small number of controls with regular morphology. The distribution of the immunostained cells in crypt epithelium and the count of stained cells evaluated with image analysis software were comparable with normal controls in all studied proteins, except for a slight increase in p21 counts in colonic mucosa in our case. These data, even if the number of control cases is restricted, suggest that proliferative activity of differentiated enterocytes of enteric mucosa is downregulated by the concurrent activity of p21 and p27. To our knowledge, the only available survey about the expression of these proteins in enteric mucosa is from authors that demonstrated the development of a phenotype with decreased expression of p27 in gastric mucosa of patients with *Helicobacter Pylori* infection (28). The FISH technique demonstrated that the gene of EGF-R, a receptor that plays a pivotal role in GLP-2 repair function toward the enteric epithelium, enhancing its activity, was regularly expressed in our case. To our knowledge, whereas data are relative to a single case, no other complete report on morphological and molecular features of mucosa attributable to neoplastic evolution was previously conducted on the patients treated with residual enteric tract of GLP-2.

In conclusion, our report is coherent with the previous surveys conducted both in pediatric (29, 30) and adult patients (mainly resected for inflammatory bowel disease) (31), in favor of the safety of the treatment with GLP-2 in SBS. An increasing evidence about the security of treatment with this drug could help in delineating the follow-up protocols. Furthermore, it must be outlined that further studies, conducted on pre- and post-treatment sampling, are mandatory for a better knowledge of teduglutide activity in restoring SBS mucosal damage.

## Data Availability Statement

The raw data supporting the conclusions of this article will be made available by the authors, without undue reservation.

## Author Contributions

EF and GCa conceived and designed the study, conducted the study, performed data collection and analysis, and wrote the manuscript. AL contributed to the study conception and design and critically reviewed the manuscript. LV and GCe contributed to the data collection and critically reviewed the manuscript. PC, CR, AO, and MS critically reviewed the manuscript. All authors contributed to the article and approved the submitted version.

## Conflict of Interest

The authors declare that the research was conducted in the absence of any commercial or financial relationships that could be construed as a potential conflict of interest.

## Publisher’s Note

All claims expressed in this article are solely those of the authors and do not necessarily represent those of their affiliated organizations, or those of the publisher, the editors and the reviewers. Any product that may be evaluated in this article, or claim that may be made by its manufacturer, is not guaranteed or endorsed by the publisher.

## References

[1] Goulet O Ruemmele F Lacaille F Colomb V. Irreversible intestinal failure. *J Pediatr Gastroenterol Nutr.* (2004) 38:250–69. 10.1097/00005176-200403000-00006 15076623

[2] D’Antiga L Goulet O. Intestinal Failure in children: the European view. *J Pediatr Gastroenterol Nutr.* (2013) 56:118–26. 10.1097/MPG.0b013e318268a9e3 22820123

[3] Drucker DJ Erlich P Asa SL Brubaker PL. Induction of intestinal epithelial proliferation by glucagon-like peptide 2. *Proc Natl Acad Sci USA.* (1996) 93:7911–6. 10.1073/pnas.93.15.7911 8755576 PMC38848

[4] Jeppesen PB Hartmann B Thulesen J Graff J Lohmann J Hansen BS Glucagon-like peptide 2 improves nutrient absorption and nutritional status in short-bowel patients with no colon. *Gastroenterology.* (2001) 120:806–15. 10.1053/gast.2001.22555 11231933

[5] Marier JF Beliveau M Mouksassi MS Shaw P Cyran J Kesavan J Pharmacokinetics, safety, and tolerability of teduglutide, a glucagon-like peptide-2 (Glp-2) analog, following multiple ascending subcutaneous administrations in healthy subjects. *J Clin Pharmacol.* (2008) 48:1289–99. 10.1177/0091270008320605 18974283

[6] Jeppesen PB Sanguinetti EL Buchman A Howard L Scolapio JS Ziegler TR Teduglutide (Alx-0600), a dipeptidyl peptidase iv resistant glucagon-like peptide 2 analogue, improves intestinal function in short bowel syndrome patients. *Gut.* (2005) 54:1224–31. 10.1136/gut.2004.061440 16099790 PMC1774653

[7] Jeppesen PB Gilroy R Pertkiewicz M Allard JP Messing B O’Keefe SJ. Randomised placebo-controlled trial of teduglutide in reducing parenteral nutrition and/or intravenous fluid requirements in patients with short bowel syndrome. *Gut.* (2011) 60:902–14. 10.1136/gut.2010.218271 21317170 PMC3112364

[8] Tappenden KA Edelman J Joelsson B. Teduglutide enhances structural adaptation of the small intestinal mucosa in patients with short bowel syndrome. *J Clin Gastroenterol.* (2013) 47:602–7. 10.1097/MCG.0b013e3182828f57 23426461

[9] Iakoubov R Lauffer LM Trivedi S Kim YI Brubaker PL. Carcinogenic effects of exogenous and endogenous glucagon-like peptide-2 in azoxymethane-treated mice. *Endocrinology.* (2009) 150:4033–43. 10.1210/en.2009-0295 19497974

[10] Trivedi S Wiber SC El-Zimaity HM Brubaker PL. Glucagon-like peptide-2 increases dysplasia in rodent models of colon cancer. *Am J Physiol Gastrointest Liver Physiol.* (2012) 302:G840–9. 10.1152/ajpgi.00505.2011 22323126

[11] Thulesen J Hartmann B Hare KJ Kissow H Orskov C Holst JJ Glucagon-like peptide 2 (GLP-2) accelerates the growth of colonic neoplasms in mice. *Gut.* (2004) 53:1145–50. 10.1136/gut.2003.035212 15247183 PMC1774162

[12] Armstrong D Forbes A Jeppesen PB Lee HM Nagy P Seidner DL. Colon polyps in patients with short bowel syndrome before and after teduglutide: post hoc analysis of the steps study series. *Clin Nutr.* (2020) 39:1774–7. 10.1016/j.clnu.2019.08.020 31522784

[13] Thymann T Stoll B Mecklenburg L Burrin DG Vegge A Qvist N Acute effects of the glucagon-like peptide 2 analogue, teduglutide, on intestinal adaptation in short bowel syndrome. *J Pediatr Gastroenterol Nutr.* (2014) 58:694–702. 10.1097/MPG.0000000000000295 24399211

[14] Kitchen PA Goodlad RA FitzGerald AJ Mandir N Ghatei MA Bloom SR Intestinal growth in parenterally-fed rats induced by the combined effects of glucagon-like peptide 2 and epidermal growth factor. *JPEN J Parenter Enteral Nutr.* (2005) 29:248–54. 10.1177/0148607105029004248 15961680

[15] Feng Y Demehri FR Xiao W Tsai YH Jones JC Brindley CD Interdependency of Egf and Glp-2 signaling in attenuating mucosal atrophy in a mouse model of parenteral nutrition. *Cell Mol Gastroenterol Hepatol.* (2017) 3:447–68. 10.1016/j.jcmgh.2016.12.005 28462383 PMC5403977

[16] Lim DW Levesque CL Vine DF Muto M Koepke JR Nation PN Synergy of glucagon-like peptide-2 and epidermal growth factor coadministration on intestinal adaptation in neonatal piglets with short bowel syndrome. *Am J Physiol Gastrointest Liver Physiol.* (2017) 312:G390–404. 10.1152/ajpgi.00281.2016 28104586

[17] Fesler Z Mitova E Brubaker PL. Glp-2, Egf, and the intestinal epithelial Igf-1 receptor interactions in the regulation of crypt cell proliferation. *Endocrinology.* (2020) 161:bqaa040. 10.1210/endocr/bqaa040 32147716 PMC7098877

[18] Gerdes J Schwab U Lemke H Stein H. Production of a mouse monoclonal antibody reactive with a human nuclear antigen associated with cell proliferation. *Int J Cancer.* (1983) 31:13–20. 10.1002/ijc.2910310104 6339421

[19] Scholzen T Gerdes J. The Ki-67 protein: from the known and the unknown. *J Cell Physiol.* (2000) 182:311–22. 10.1002/(SICI)1097-4652(200003)182:33.0.CO;2-9 10653597

[20] Jurikova M Danihel L Polak S Varga I. Ki67, Pcna, and Mcm proteins: markers of proliferation in the diagnosis of breast cancer. *Acta Histochem.* (2016) 118:544–52. 10.1016/j.acthis.2016.05.002 27246286

[21] Kreis NN Louwen F Yuan J. The multifaceted P21 (Cip1/Waf1/Cdkn1a) in cell differentiation, migration and cancer therapy. *Cancers.* (2019) 11:11091220. 10.3390/cancers11091220 31438587 PMC6770903

[22] Razavipour SF Harikumar KB Slingerland JM. P27 as a transcriptional regulator: new roles in development and cancer. *Cancer Res.* (2020) 80:3451–8. 10.1158/0008-5472.CAN-19-3663 32341036

[23] Schindelin J Rueden CT Hiner MC Eliceiri KW. The imagej ecosystem: an open platform for biomedical image analysis. *Mol Reprod Dev.* (2015) 82:518–29. 10.1002/mrd.22489 26153368 PMC5428984

[24] Villanacci V Ceppa P Tavani E Vindigni C Volta U. Gruppo Italiano Patologi Apparato D et al Coeliac Disease: the histology report. *Dig Liver Dis.* (2011) 43:S385–95. 10.1016/S1590-8658(11)60594-X 21459344

[25] Das P Gahlot GP Singh A Baloda V Rawat R Verma AK Quantitative histology-based classification system for assessment of the intestinal mucosal histological changes in patients with celiac disease. *Intest Res.* (2019) 17:387–97. 10.5217/ir.2018.00167 30996219 PMC6667359

[26] Adelman DC Murray J Wu TT Maki M Green PH Kelly CP. Measuring change in small intestinal histology in patients with celiac disease. *Am J Gastroenterol.* (2018) 113:339–47. 10.1038/ajg.2017.480 29460921

[27] Taavela J Koskinen O Huhtala H Lahdeaho ML Popp A Laurila K Validation of morphometric analyses of small-intestinal biopsy readouts in celiac disease. *PLoS One.* (2013) 8:e76163. 10.1371/journal.pone.0076163 24146832 PMC3795762

[28] Shirin H Sordillo EM Kolevska TK Hibshoosh H Kawabata Y Oh SH Chronic *Helicobacter Pylori* infection induces an apoptosis-resistant phenotype associated with decreased expression of P27(Kip1). *Infect Immun.* (2000) 68:5321–8. 10.1128/IAI.68.9.5321-5328.2000 10948161 PMC101795

[29] Naberhuis JK Tappenden KA. Teduglutide for safe reduction of parenteral nutrient and/or fluid requirements in adults: a systematic review. *JPEN J Parenter Enteral Nutr.* (2016) 40:1096–105. 10.1177/0148607115582063 25883117

[30] Pironi L Raschi E Sasdelli AS. The safety of available treatment options for short bowel syndrome and unmet needs. *Expert Opin Drug Saf.* (2021) 20:1501–13. 10.1080/14740338.2021.1940947 34105428

[31] Kochar B Long MD Shelton E Young L Farraye FA Yajnik V Safety and efficacy of teduglutide (gattex) in patients with crohn’s disease and need for parenteral support due to short bowel syndrome-associated intestinal failure. *J Clin Gastroenterol.* (2017) 51:508–11. 10.1097/MCG.0000000000000604 27433811 PMC5243925

