# Supplementary material for: Case Report: Morphologic and Functional Characteristics of Intestinal Mucosa in a Child With Short Bowel Syndrome After Treatment With Teduglutide: Evidence in Favor of GLP-2 Analog Safety
Source: Front Nutr. 2022 Jun 23;9:866048. doi: 10.3389/fnut.2022.866048 (PMC9261410; doi:10.3389/fnut.2022.866048)
Supplement: Supplementary file 1 [file Data_Sheet_1.pdf]

CARE case report guidelines

CARE Checklist of information to include when writing a case report

(cc) BY-NC-ND

Topic	Item	Checklist item description	Reported on Line
Title	1	The diagnosis or intervention of primary focus followed by the words "case report"	line 65
Key Words	2	2 to 5 key words that identify diagnoses or interventions in this case report, including "case report"	line 180
Abstract (no references)	3a	Introduction: What is unique about this case and what does it add to the scientific literature?	line 176-178
	3b	Main symptoms and/or important clinical findings	line 102-176
	3c	The main diagnoses, therapeutic interventions, and outcomes	line 98-100
	3d	Conclusion—What is the main "take-away" lesson(s) from this case?	line 176-178
Introduction	4	One or two paragraphs summarizing why this case is unique (**may include references**)	line 214-217
Patient Information	5a	De-identified patient specific information.	yes
	5b	Primary concerns and symptoms of the patient.	NA
	5c	Medical, family, and psycho-social history including relevant genetic information	NA
	5d	Relevant past interventions with outcomes	line 176-178
Clinical Findings	6	Describe significant physical examination (PE) and important clinical findings.	NA
Timeline	7	Historical and current information from this episode of care organized as a timeline	NA
Diagnostic Assessment	8a	Diagnostic testing (such as PE, laboratory testing, imaging, surveys).	line 221-474
	8b	Diagnostic challenges (such as access to testing, financial, or cultural)	NA
	8c	Diagnosis (including other diagnoses considered)	line 221-225
	8d	Prognosis (such as staging in oncology) where applicable	NA
Therapeutic Intervention	9a	Types of therapeutic intervention (such as pharmacologic, surgical, preventive, self-care)	line 225-287
	9b	Administration of therapeutic intervention (such as dosage, strength, duration)	line 225-287
	9c	Changes in therapeutic intervention (with rationale)	NA
Follow-up and Outcomes	10a	Clinician and patient-assessed outcomes (if available)	NA
	10b	Important follow-up diagnostic and other test results	line 225-287
	10c	Intervention adherence and tolerability (How was this assessed?)	line 239
	10d	Adverse and unanticipated events	line 239
Discussion	11a	A scientific discussion of the strengths AND limitations associated with this case report	line 796-801
	11b	Discussion of the relevant medical literature **with references**.	line 788-792
	11c	The scientific rationale for any conclusions (including assessment of possible causes)	line 793-801
	11d	The primary "take-away" lessons of this case report (without references) in a one paragraph conclusion	line 793-801
Patient Perspective	12	The patient should share their perspective in one to two paragraphs on the treatment(s) they received	NA
Informed Consent	13	Did the patient give informed consent? Please provide if requested	Yes ☒ No ☐
